# COX-2 gene expression in colon cancer tissue related to regulating factors and promoter methylation status

**DOI:** 10.1186/1471-2407-11-238

**Published:** 2011-06-13

**Authors:** Annika Gustafsson Asting, Helena Carén, Marianne Andersson, Christina Lönnroth, Kristina Lagerstedt, Kent Lundholm

**Affiliations:** 1 Department of Surgery, Institute of Clinical Sciences, Sahlgrenska Academy, Sahlgrenska University Hospital, Gothenburg, Sweden; 2 Department of Clinical Genetics, Institute of Biomedicine, University of Gothenburg, Sweden

## Abstract

**Background:**

Increased cyclooxygenase activity promotes progression of colorectal cancer, but the mechanisms behind COX-2 induction remain elusive. This study was therefore aimed to define external cell signaling and transcription factors relating to high COX-2 expression in colon cancer tissue.

**Method:**

Tumor and normal colon tissue were collected at primary curative operation in 48 unselected patients. COX-2 expression in tumor and normal colon tissue was quantified including microarray analyses on tumor mRNA accounting for high and low tumor COX-2 expression. Cross hybridization was performed between tumor and normal colon tissue. Methylation status of up-stream COX-2 promoter region was evaluated.

**Results:**

Tumors with high COX-2 expression displayed large differences in gene expression compared to normal colon. Numerous genes with altered expression appeared in tumors of high COX-2 expression compared to tumors of low COX-2. COX-2 expression in normal colon was increased in patients with tumors of high COX-2 compared to normal colon from patients with tumors of low COX-2. IL1β, IL6 and iNOS transcripts were up-regulated among external cell signaling factors; nine transcription factors (ATF3, C/EBP, c-Fos, Fos-B, JDP2, JunB, c-Maf, NF-κB, TCF4) showed increased expression and 5 (AP-2, CBP, Elk-1, p53, PEA3) were decreased in tumors with high COX-2. The promoter region of COX-2 gene did not show consistent methylation in tumor or normal colon tissue.

**Conclusions:**

Transcription and external cell signaling factors are altered as covariates to COX-2 expression in colon cancer tissue, but DNA methylation of the COX-2 promoter region was not a significant factor behind COX-2 expression in tumor and normal colon tissue.

## Background

Colorectal cancer is common in Western countries with unanimous findings that prostaglandins are important for both carcinogenesis and progression [1-3]. It has been repeatedly observed that Cyclooxygenase-1/-2 inhibition attenuates appearance of epithelial cancer in experimental models and in part also in patients. Both primary and secondary prevention with cyclooxygenase (COX) inhibitors demonstrate and confirm decreased incidence of colorectal carcinoma in both retrospective and randomized patient cohorts [4-7]. Thus, a large number of observations emphasize that induced prostaglandin production, particularly PGE_2_, is involved in cell signaling through prostanoid receptors, where our own observations suggested subtype EP_2 _receptor expression in colon cancer tissue to predict reduced survival [2]. An important issue behind the appearance and progression of colorectal cancer seems to be increased production of PGE_2 _secondary to COX-2 induction in both transformed epithelial cells and host stroma [2,4,8-11]. Mechanisms behind COX-2 gene induction in colorectal cancer disease remain elusive, although a lot of information is available on the regulation of COX-2 gene expression [12,13]. Most such information has, however, been obtained in isolated cell culture experiments with obvious limitations compared to the in vivo situation based on uninterrupted host and tumor cell signaling. Therefore, the aim of the present study was to relate well-recognized external cell- and transcription factor expression to elevated tumor COX-2 expression in colorectal cancer tissue at primary operations aimed at cure.

## Methods

### Patients

**Figure 1:**
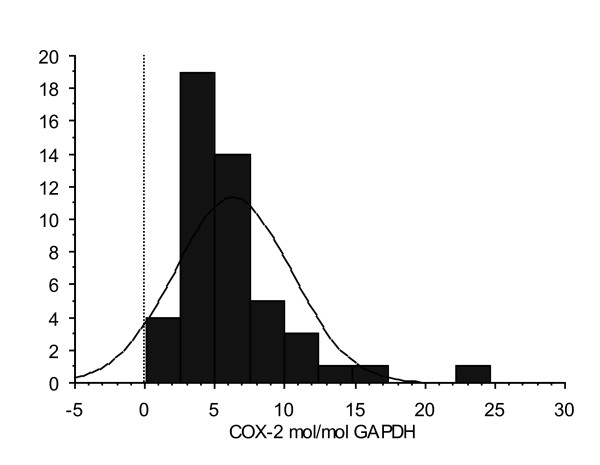
**Distribution of COX-2 among patients (n = 48)**. COX-2 expressions of tumors used in this study were distributed at the top and bottom quartile.

Tumor and colon tissue samples were collected from 48 unselected patients at primary operation for colorectal carcinoma between 2001 to 2004 in Uddevalla county of Sweden. All patients underwent surgery as the only curative treatment and none received neoadjuvant radio-chemotherapy, according to individual decisions and institutional indications. Normal colon tissue was collected as a minimum of 10 cm away from the macroscopically seen tumor tissue. The group of patients consisted of 54% males and 46% females with a mean age of 72.5 years (range 40 to 91 years) at surgery. Mean survival time was 27.3 ± 4.2 months (range 0 to 73 months) following surgery according to a recent update of survival (Dec 2008), where 21 patients were still alive. Tumors were histologically classified as Dukes A (8), Dukes B (19), Dukes C (11) and Dukes D (10). PCR analysis was performed to quantify COX-2 mRNA expression [2]. Mean COX-2 expression in tumor tissue for the entire group was 6.27 ± 0.60 mol/mol GAPDH (range 0.11 to 24.67) (n = 48) and 14.6 ± 1.7 (range 1.7 to 38.08) (n = 29) in normal colon tissue. Patients used in subsequent experiments were selected according to COX-2 expression in tumors; one group of 10 patients with highest and one group of 10 patients with lowest COX-2 expression (Figure 1, Table 1).

**Table 1:** Classification of patients with high or low COX-2 expression in tumor tissue

	COX-2 expression in tumor tissue	
	**High (10)**	**Low (10)**

COX-2 T	12.5 ± 1.5	2.6 ± 0.4
(mol/mol GAPDH) NM	5.06 ± 3.0	12.3 ± 4.0

Dukes^1^	3 A, 6 B, 1 D	3 B, 5 C, 2 D

Age at surgery	71 ± 2.5	73 ± 3.4

Gender	6 M, 4 F	4 M, 6 F

Postoperative survival (months)	23 ± 5.8	29 ± 8.0

Right/left colon	4/6	4/6

Mean ± SEM

T = tumor tissue

NM = Normal colon tissue

^1^Approx. TNM; A = T1, N0, M0; B = T2/T3, N0, M0; C = T2/T3/T4, N1/N2, M0; D = T2/T3/T4, N1/N2, M1

### RNA and DNA extraction

Tumor and normal colon tissue samples were collected down to the serosa layer during surgery and kept fresh frozen in liquid nitrogen and stored in -80°C until analysis. Certified pathologists staged all tumors. Tumor samples were secured by the surgeon in charge and contained around 55-75% tumor cells according to random visual inspection. RNA extraction and cDNA synthesis were performed as described [2]. A quality check of RNA in all samples was done in Bioanalyzer 2100 with limit RIN 6.0 for further analysis (Agilent). DNA was extracted with QIAamp^® ^DNA Mini Kit (Qiagen) according to instructions. RNase A solution (Promega) was used as extra step in DNA purification. Concentration of RNA and DNA was determined in NanoDrop^® ^ND-1000 Spectrophotometer (NanoDrop Technologies).

### Gene expression analysis

Microarray analysis was performed on pooled tumor mRNA from 9 out of 10 patients with high COX-2 mRNA expression versus 9 out of 10 patients with low COX-2 mRNA expression. The two patients were excluded due to low RIN indicating poor RNA quality. 500 ng of mRNA from tumors with high COX-2 patients was labeled with Cy-3-dCTP (Amersham BioSciences) in a cDNA synthesis reaction with Agilent Flourescent Direct Label. Tumor samples with low COX-2 expression (500 ng) were labeled with Cy-5-dCTP. Pooled tumor cDNA from patients with high COX-2 versus pooled DNA from patients with low COX-2 expression were then hybridized on whole human oligo genome microarrays (4 × 44K expression arrays, Agilent) during 18 hrs followed by post-hybridization washes according to in situ instructions (Hybridization Kit Plus, Agilent). Microarrays were quantified on Agilent G2565 AA microarray scanner and data were pre-processed in Feature Extraction 9.1 software program (Agilent). The same procedures were performed with normal colon mucosa tissue from all patients with only 4 patients per pool due to poor RNA quality. One hybridization with high COX-2 tumor expression versus corresponding normal colon mucosa tissue was also performed. Four technical replicates of all tumor tissue analyses and two technical replicates of normal colon tissue and tumor versus normal colon tissue analyses were performed. Dye-normalized, outlier- and background subtracted values were analyzed in GeneSpring GX 10 software program (Agilent) according to standard procedures. A quality control was performed with QC metrics. Analysis used were filter on flags (present or marginal) were 27194 probes (out of 41078) passed in 2 out of 4 technical replicates with tumor tissue. In normal tissue 25735 probes passed and in tumor vs normal tissue 27919 probes passed filter on flags for further analysis with t-test (p < 0.05). Fold change 1.5 of log2 transformed ratios was considered a statistically significant change in gene expression. Significant pathway analysis with entity list FC 1.5 was used to define significantly altered pathways. PCR analyses were performed at Tataa Biocenter (Gothenburg, Sweden) in a LightCycler 480 Probe Master (Roche). PCR assays (AKT1 HS00178289_m1, IL1B HS01555410_m1, IL6 HS00985639_m1, TRA@ HS 00612292_m1, CARD11 HS01060626_m1, Applied Biosystems) were tested and validated for efficiency and specificity. All samples were run in duplicate and negative controls were negative. Results were related to assay efficiency and GAPDH (GAPDH assay from Tataa Biocenter's Reference Gene Panel Human) and calculated according to comparative Ct method.

### DNA methylation analysis with bisulfite sequencing

Methylation analysis was performed with tag-modified bisulfite genomic sequencing [14]. EpiTect^® ^Bisulfite kit (Qiagen) was used for bisulphite modification of 1 μg tumor and normal colon tissue DNA from each patient (n = 20) according to instructions. Modified DNA samples were amplified with two different sets of primers (Table 2) directed towards two areas of the COX-2 promoter region using touchdown PCR (1 × Reaction Buffer, 0.5 mM dNTPs, 2.0 mM MgCl_2_, 0.4 μM forward and reverse primers resp., and 1 unit HotStart Taq (Qiagen). Primer pair 1 was taken from Hur et al. and primer pair 2 was designed with BiSearch [15,16]. PCR reactions were denatured at 95°C for 10 min, then 20 cycles of 95°C 45 s, 60°C/65°C annealing temperature with a decrease of half a degree per cycle for 45 s, 68°C 60 s followed by 15-20 cycles of 95°C 45 s, 50°C/55°C 45 s and 68°C 60 s ended with a 7 min extension at 68°C. EpiTect^® ^PCR Controls (Qiagen) was used to ensure that the reaction worked properly; one methylated control sample, one unmethylated control and a 50/50 mixture of methylated and unmethylated controls were included in each PCR to ensure that methylated and unmethylated template were both equally amplified. The specificity of PCR products was inspected by use of 2100 Bioanalyzer according to protocol for DNA 1000 (Agilent). PCR products were then purified using Agencourt AMPure magnetic beads (Agencourt Bioscience Corporation) using the Biomek NX pipetting robot (Beckman Coulter) and eluted in distilled water. Sequence PCR was performed using forward or reverse primer with the ABI Prism BigDye™ cycle sequencing Ready Reaction Kit v1.1 (Applied Biosystems). Sequence PCR was run in 10 μl reactions under following conditions: 96°C 1 min, followed by 25 cycles of 96°C 10 s and 50°C 4 min. Sequencing products were purified using CleanSeq magnetic beads (Agencourt) using the Biomek NX and re-suspended in 10 μl of High Dye formamide (Applied Biosystems). The sequencing products were separated with gel electrophoresis on a 3730 DNA analyser (Applied Biosystems) and the output data were viewed and analysed using Sequence Analysis v5.2 (Applied Biosystems) and BiqAnalyzer [17].

**Table 2:** Primers used for methylation analysis

	Tag	Primer Sequence (5'-3')	Bp
1	FP1692	**CCACTCACTCACCCACCC**GAAGAAGAAAAGATATTTGG	448

1	RP2104	**GGGTGGGAGGTGGGAGGG**ATAAACTTTACTATCTAAAA	

2	B1(1595)	**CCACTCACTCACCCACCC**TTGGAGAGGAAGTTAAGTGTTT	223

2	b1(1782)	**GGGTGGGAGGTGGGAGGG**ATCCCCACTCTCCTATCTAAT	

### Statistics

Results are presented as mean ± SEM. Statistical analyses were performed by ANOVA and p < 0.05 was regarded significant in two-tailed tests. This study was approved by the board of Ethics at University of Gothenburg (NCT00473980). Accordingly, all patients participated with informed consent.

## Results

### COX-2 expression

Large difference in gene expression was observed when comparing tumor tissue with high COX-2 expression to normal colon mucosa tissue from the same patients (2557↑, 3182↓) (Table 3). A large number of genes with altered expression appeared also in colon cancer tissue of tumors with high COX-2 expression when compared to tumors with low COX-2 expression (3086↑, 3031↓) (Table 4). Expression of COX-2 in normal colon mucosa tissue was significantly increased in patients with tumors of high COX-2 expression compared with mucosa from patients with low COX-2 expression in tumors (776↑, 804↓). Highly expressed genes in tumor tissue with high COX-2 expression were associated to cell motility, cell structure, muscle proteins, and energy homeostasis while down-regulated genes in such tumors seemed to be related to tumor antigens as Melanoma antigen and Chondrosarcoma ass. Gene, etc (Table 4). Several factors of immune response as serpin peptidase inhibitor and inducible metric oxide synthase 2 with antitumoral activities were either up- or down-regulated in normal colon tissue from patients with tumors of high COX-2 expression (Table 5). Patients with tumors with high COX-2 expression did not in the present material display significantly reduced survival compared to patients with tumors with low COX-2 expression (Table 1). However, COX-2 expression predicted survival at borderline significance in a larger patient material [2].

**Table 3:** Genes listed according to the magnitude of most altered expression between tumor tissue and normal mucosa from the same patients with tumors of high intrinsic COX-2 expression

Gene	↑/↓	FC	Function	Gene Symbol (GeneID)
Matrix metallopeptidase 7	**↑**	110	Breakdown of extracellular matrix, metastasis	MMP7 (4316)

Keratin 23	**↑**	77	Structural integrity of epithelial cells	KRT23 (25984)

Myosin	**↑**	63	Muscle, heavy polypeptide 2	MYH2 (4620)

Claudin 1	**↑**	38	Integral membrane protein, component of tight junction strands	CLDN1 (9076)

Actin α1, skeletal	**↑**	36	Cell motility, structure and integrity	ACTA1 (58)

Forkhead box Q1	**↑**	34	Transcription factor (i.e. TGF-beta2)	FOXQ1 (94234)

Proprotein convertase subtilisin/kexin type 1	**↑**	22	Regulating insulin biosynthesis, obesity, activate precursor protein, associated with carcinoid tumors	PCSK1 (5122)

Sarcolipin	**↑**	25	Regulates several sarcoplasmic reticulum Ca(2+)-ATPases	SLN (6588)

Matrix metallopeptidase 1	**↑**	14	Breakdown of extracellular matrix, metastasis	MMP1 (4312)

Myosin, cardiac, beta	**↑**	25	Heavy chain, also expressed in skeletal muscle tissues rich in slow-twitch type I muscle fibers	MYH7 (4625)

Hydroxy-δ-5-steroid dehydrogenase	**↓**	79	Biosynthesis of steroid hormones, polymorphisms related to prostate cancer	HSD3B2 (3284)

Hydroxy-δ-5-steroid dehydrogenase	**↓**	30	Biosynthesis of steroid hormones, diagnostic trophoblastic marker	HSD3B1 (3283)

similar to 3 β-hydroxysteroid dehydrogenase	**↓**	28	Pseudogene	LOC391081 (391081)

-	**↓**	24	unknown	CR627415

Butyrylcholinesterase	**↓**	21	Homozygoutics sustain prolonged apnea after administration of the muscle relaxant suxamethonium	BCHE (590)

Neuronal pentraxin	**↓**	19	Excitatory synapse remodelling, mediate neuronal death induced by reduction in neuronal activity in mature neurons	NPTX1 (4884)

Dipeptidyl-peptidase 6	**↓**	18	Binds and alters specific voltage-gated potassium channels expression and biophysical properties	DPP6 (1804)

ATP-binding cassette, sub-family G	**↓**	17	Breast cancer resistance protein, xenobiotic transporter may play a major role in multi-drug resistance	ABCG2 (9429)

Insulin like-5	**↓**	17	Relaxin/Insulin family, ligand for GPCR142	INSL5 (10022)

Regenerating islet-derived 1 β	**↓**	16	Highly similar to REG1A protein (islet cell regeneration and diabetogenesis)	REG1B (5968)

**↑ **increased expression

**↓ **decreased expression

**Table 4:** Genes listed according to the magnitude of most altered expression in tumor tissue of high intrinsic COX-2 expression

Gene	↑/↓	FC	Function	Gene Symbol (GeneID)
Unknown	**↑**	156	unknown	W60781

Myosin	**↑**	107	Muscle, heavy polypeptide 2	MYH2 (4620)

Desmin	**↑**	49	Muscle filament	DES (1674)

Creatine kinase	**↑**	46	Muscle, energy homeostasis, serum marker for myocardial infarction	CKM (1158)

Troponin T type 3	**↑**	41	Skeletal	TNNT3 (7140)

Actin α, cardiac	**↑**	37	Muscle, contractile, cell motility	ACTC1 (70)

Actin α1, skeletal	**↑**	35	Cell motility, structure and integrity	ACTA1 (58)

Synaptopodin 2	**↑**	28	Cell-shape regulation, homolog with Myopodin (a tumor suppressor; inhibits growth and metastasis)	SYNPO2 (171024)

Nebulin	**↑**	25	Cytoskeletal matrix	NEB (4703)

Calponin	**↑**	23	Differentiation marker, reduced in tumor vessels associated with tumor progression	CNN1 (1264)

Growth diff. factor 10	**↓**	28	Regulation of cell growth and differentiation	GDF10 (2662)

Unc-13 homolog A	**↓**	19	diacylglycerol and phorbol ester receptors, essential role in synaptic vesicle priming	UNC13A (23025)

LOC553137	**↓**	16	miscRNA, unknown function	LOC553137 (553137)

Melanoma antigen family B, 2	**↓**	14	Tumor-associated antigen, expressed only in testis and tumors, regulated by demethylation	MAGEB2 (4113)

Chondrosarcoma associated gene 1	**↓**	13	Tumor antigen, expressed in testis and in chondrosarcomas	CSAG1 (158511)

Folate receptor 1	**↓**	13	Involvement in cancer prognosis	FOLR1 (2348)

Renin	**↓**	13	Blood pressure and electrolyte balance	REN (5972)

Small proline-rich protein 3	**↓**	12	Epithelial homeostatis, aberrant expression contribute to tumorigenesis of esophageal squamous cell carcinoma	SPRR3 (6707)

Heat shock 27kDa protein 3	**↓**	12	Muscle	HSPB3 (8988)

Chromogranin B	**↓**	11	Precursor for biological active peptides, involved early in breast cancer	CHGB (1114)

**↑ **increased expression

**↓ **decreased expression

**Table 5:** Genes listed according to the magnitude of most altered expression in normal mucosa from patients with tumors of high intrinsic COX-2 expression

Gene	↑/↓	FC	Function	Gene Symbol (GeneID)
Pancreatic derived factor (PANDER)	**↑**	79	Cytokine, induces apoptosis of alpha and beta cells, implicated in diabetes	FAM3B (54097)

Regenerating islet-derived 1 α	**↑**	40	Secreted by the exocrine pancreas, associated with islet cell regeneration and diabetogenesis	REG1A (5967)

Ribosomal protein S4, Y-linked 2	**↑**	40	Translation	RPS4Y2 (140032)

Serpin peptidase inhibitor, ovalbumin	**↑**	35	Cytoprotective, cell survival factor, monocyte regulation, potential cancer marker (elevated in plasma)	SERPINB2 (5055)

Nitric oxide synthase 2, inducible	**↑**	19	reactive free radical, biologic mediator in several processes, incl. neurotransmission, antimicrobial and antitumoral activities	NOS2A (4843)

Pentraxin-related gene	**↑**	16	Immune response, inflammation, rapidly induced by IL-1β	PTX3 (5806)

Regenerating islet-derived 1 β	**↑**	16	highly similar to REG1A protein	REG1B (5968)

Hydroxy-delta-5-steroid dehydrogenase	**↑**	10	Biosynthesis of steroid hormones, polymorphisms related to prostate cancer	HSD3B2 (3284)

Hydroxy-delta-5-steroid dehydrogenase	**↑**	10	Biosynthesis of steroid hormones, diagnostic trophoblastic marker	HSD3B1 (3283)

ADAM metallopeptidase	**↑**	10	cleavage of proteoglycans, control of organ shape during development, and inhibition of angiogenesis	ADAMTS9 (56999)

Unknown	**↓**	15	unknown	ENST00000343

6-phosphofructo-2-kinase	**↓**	13	Produce fructose 2,6-P(2), involved in Warburg effect	PFKFB3 (5209)

hemoglobin, γ A	**↓**	8	Normally expressed in the fetal liver, spleen and bone marrow	HBG1 (3047)

matrix metallopeptidase 7	**↓**	8	Breakdown of extracellular matrix, metastasis	MMP7 (4316)

Fc receptor-like 4	**↓**	7	Immune regulation	FCRL4 (83417)

Chemokine receptor 5	**↓**	6	B cell migration and localization	BLR1 (643)

Fibroblast growth factor 5	**↓**	6	Mitogenic and cell survival activities, involved in a variety of biological processes, incl. cell growth, morphogenesis, tissue repair, tumor growth and invasion, oncogene	FGF5 (2250)

Family with sequence similarity 129, member C	**↓**	6	B-cell novel protein, BCNP1 completely unknown protein with 3 predicted transmembrane domains	FAM129C (199786)

selectin L	**↓**	6	Leukocyte-endothelial cell interactions	SELL (6402)

Zinc finger protein	**↓**	5	DNA binding	ZNF683 (257101)

**↑ **increased expression

**↓ **decreased expression

### Transcription factors

A large number of transcription factors with reported importance for regulation of the COX-2 gene in human cells were evaluated and are listed in Table 6. Eight of these transcription factors showed increased expression, while 5 transcription factors were down-regulated in tumors with high COX-2 expression.

**Table 6:** Significant alterations in expression of previously reported important transcription factors in tumors of high intrinsic COX-2 expression

Gene	Product	FC 1.5	FC 2.0	FC 3.0	Reference^a^
TFAP2C	AP-2γ	**↓**	**-**	-	[12]

TFAP2E	AP-2ε	**-**	**-**	-	[12]

ATF1	ATF1	**-**	**-**	-	[13]

ATF2	ATF2/CREB2	**-**	**-**	-	[13]

ATF3	ATF3/AP1	**↑**	**-**	-	[13]

BATF	B-ATF	**-**	**-**	-	[13]

CREBBP	CBP/p300 co-activator	**-**	**-**	-	[12]

CDX2	CDX2	**↓**	**-**	-	[29]

CEBPA	C/EBP-α	**-**	**-**	-	[13]

CEBPB	C/EBP-β	**-**	**-**	-	[13]

CEBPD	C/EBP-δ	**↑**	**↑**	-	[13]

CREB5	CRE-BPA	**-**	**-**	-	[13]

CREB1	CREB	**-**	**-**	-	[13]

ELK1	Elk-1	**↓**	**-**	-	[13]

FOS	c-Fos/AP1	**↑**	**-**	-	[12]

FOSB	Fos-B/AP1	**↑**	**↑**	**↑**	[12]

FOSL1	Fra-1/AP1	**-**	**-**	-	[12]

FOSL2	Fra-2/AP1	**-**	**-**	-	[12]

DNAJC12	JDP1	**-**	**-**	-	[12]

JDP2	JDP2	**↑**	**-**	-	[12]

JUN	Jun/AP1	**-**	**-**	-	[12]

JUNB	JunB/AP1	**-**	**-**	-	[12]

JUND	JunD/AP1	**↑**	**-**	-	[12]

MAF	c-Maf/AP1	**↑**	**↑**	-	[12]

NFATC1	NFATc1/NFAT2	**-**	**-**	-	[12]

NFATC4	NFAT3	**-**	**-**	-	[12]

NFATC3	NFAT4	**-**	**-**	-	[12]

NFAT5	NFAT5	**-**	**-**	-	[12]

NFKB1	NF-κB, p105/p50	**-**	**-**	-	[12,13]

NFKB2	NF-κB, p100	**-**	**-**	-	[12,13]

RELA	NF-κB, RelA (p65)	**-**	**-**	-	[12,13]

RELB	NF-κB, RelB	**-**	**-**	-	[12,13]

REL	NF-κB, c-Rel	**-**	**-**	-	[12,13]

NFKBIA	NF-κB, IκBα	**↑**	**↑**	-	[12,13]

NFKBIB	NF-κB, IκBβ	**-**	**-**	-	[12,13]

NFKBIE	NF-κB, IκBε	**-**	**-**	-	[12,13]

CHUK	NF-κB, IKK-α	**-**	**-**	-	[12,13]

IKBKB	NF-κB, IKK-β	**-**	**-**	-	[12,13]

IKBKG	NF-κB, IKK-γ	**-**	**-**	-	[12,13]

TP53	p53	**↓**	**↓**	-	[30]

ETV4	PEA3	**↓**	**-**	-	[13]

PPARA	PPARα	**-**	**-**	-	[12]

PPARD	PPARβ/δ	**-**	**-**	-	[12]

PPARG	PPARγ	**-**	**-**	-	[12]

SP1	SP-1	**-**	**-**	-	[12]

TBP	TATA-binding protein	**-**	**-**	-	[12]

TCF4	TCF4	**↑**	**↑**	-	[13]

AP-2α, AP-2β, AP-2δ, NFAT1 and Retinoic acid receptor were also tested, but no expression were found.

**↑ **increased expression; **↓ **decreased expression; FC is log2 fold changes

^a ^published report with emphasis on transcription factor in regulation of COX-2 gene.

### External factors

Genes with reported functions in external cell signaling indicated 6 genes with increased expression, while no gene indicated down-regulation related to high COX-2 expression in tumor tissue (Table 7). Table 8 describes genes with significantly altered expression of transcription factors and external cell signaling factors in normal mucosa from patients with tumors of increased COX-2 expression. IL1-β, IL6, and iNOS were up-regulated, probably inducing a variety of transcription factors.

**Table 7:** Significant alterations in expression of previously reported important external cell signaling factors and enzymes in tumors of high intrinsic COX-2 expression

Gene	Product	FC 1.5	FC 2.0	FC 3.0	Reference^a^
DNMT1	DNA MTase 1	**-**	-	-	[26]

DNMT3A	DNA MTase 3A	**↑**	**↑**	-	[26]

DNMT3B	DNA MTase 3B	**-**	-	-	[26]

MDM2	hdm2	**-**	-	-	[30]

HPGD	HPGD	**-**	-	-	[1]

HNRPD	HuR	**-**	-	-	[31]

IL1B	IL1b	**↑**	**↑**	-	[32,33]

IL6	IL6	**↑**	**↑**	**↑**	[34]

IL6R	IL6 receptor	**↑**	-	-	[34]

NOS2A	iNOS	**-**	-	-	[13]

MAPK14	p38 MAPK	**-**	-	-	[35]

PTGES	mPGES-1	**-**	-	-	[1]

PRKCB1	Protein kinas Cβ1	**↑**	**↑**	**↑**	[12]

RALA	Ras family (oncogene)	**-**	-	-	[12]

RASA4	Ras p21 protein activator	**-**	-	-	[12]

RASSF1	Ras ass. (tumor supr.)	**-**	-	-	[12]

RIN2	Ras and Rab interact. 2	**-**	-	-	[12]

TINP1	TGF-β	**-**	-	-	[13]

TNF	TNF-α	**↑**	-	-	[13]

**↑ **increased expression

**↓ **decreased expression

FC is log2 fold change

^a ^published report with emphasis on the current factors

**Table 8:** Significant alterations in expression of transcription factors and cell signaling factors in normal colon mucosa from patients with tumors of high intrinsic COX-2 expression

Gene	Product	FC 1.5	FC 2.0	FC 3.0
PTGS2	COX-2	**↑**	**↑**	**↑**

Transcription				

ATF3	ATF3/AP1	**↑**	-	-

CEBPD	C/EBP-δ	**↑**	**↑**	-

CREB1	CREB	**↑**	**-**	-

FOSB	Fos-B/AP1	**↑**	**↑**	-

DNAJC12	JDP1	**↑**	**-**	-

NFATC1	NFATc1/NFAT2	**↓**	-	-

PPARG	PPARγ	**↓**	-	-

BATF	B-ATF	**↓**	-	-

External cell factors				

IL1B	IL1b	**↑**	**↑**	**↑**

IL6	IL6	**↑**	**↑**	**↑**

NOS2A	iNOS	**↑**	**↑**	**↑**

All factors listed in Table 6 and 7 were evaluated.

### Pathway analysis

Significantly altered metabolic and signaling pathways (FC 1.5) in tumors with high COX-2 expression were mainly related to immunity according to algorithm analyses (GeneSpring GX 10, Agilent). Five genes that were significantly changed in arrays (AKT1, CARD11, IL1B, IL6, and TRA@) and involved in significantly changed pathways according to the algorithm analyses (TCR, BCR, IL1, and IL6) were tested individually (Table 9). All results from individual PCR analyses agreed with array results with one exception; in tumor tissue with high compared to low COX-2 expression AKT1 expression was significantly higher in PCR analyses, with opposite direction in array analysis.

**Table 9:** Gene expressions in tumor tissue with high COX-2 expression compared to tumor tissue with low COX-2 expression confirmed by q-PCR

	CARD 11		TRA@		IL6		IL1B		AKT1	
	High COX-2 (9)	Low COX-2 (9)	High COX-2 (9)	Low COX-2 (9)	High COX-2 (9)	Low COX-2 (9)	High COX-2 (9)	Low COX-2 (9)	High COX-2 (9)	Low COX-2 (9)

Mean	0.40 ± 0.08	0.42 ± 0.24	2.87 ± 0.63	0.55 ± 0.14	1.97 ± 0.79	0.09 ± 0.03	1.61 ± 0.49	0.27 ± 0.06	1.09 ± 0.13	0.62 ± 0.07

Median	0.39	0.07	3.12	0.44	0.87	0.05	1.01	0.21	0.96	0.59

Variance	0.06	0.50	3.57	0.18	5.65	0.008	2.16	0.04	0.14	0.04

P-value*	ns		0.002		0.03		0.02		0.005	

*ANOVA analysis

CARD11 = caspase recruitment domain family, member 11

TRA@ = T cell receptor alpha locus

IL6 = Interleukin 6

IL1B = Interleukin 1β

AKT1 = v-akt murine thymoma viral oncogene homolog 1 or protein kinase B

### DNA methylation

A majority of tumor tissue specimens displayed no methylation of the promoter region of the COX-2 gene in tumor tissue; only 2 tumors showed methylation of the promoter region (low COX-2, Dukes B tumors with intermediate differentiation). Methylation of the promoter region of the COX-2 gene was not observed in any of the normal colon mucosa specimens.

## Discussion

Increased prostanoid activity is a well recognized characteristic of colorectal cancer [4,8,10,18-20]. Early, it was claimed that such tumors have increased COX-2 expression deduced from immunohistochemical evaluations of tumor tissue specimens. More recent investigations have however emphasized that increased COX-2 expression in colorectal cancer is preferentially a local phenomenon with uneven distribution among transformed cells without overall increased tissue content [11]. These findings imply heterogenous cell clones or that other factor are responsible for either up- or down-regulation of COX-2 in malignant cells. It is also important to underline that increased COX-2 expression in tumor tissue may well be confined to stroma and host cells. Such a complex condition supports cross-talk among cells with prostanoids as signals through specific receptors. Our previous investigations have demonstrated a statistically significant relationship between COX-2 expression and tumor tissue production of PGE_2_, which is elevated in colon cancer tissue [11]. These kinds of findings agree with information that both primary and secondary intervention with cyclooxygenase inhibitors influenced on both local tumor growth and systemic effects in experimental and clinical cancer [4]. Of particular interest may be previous observations that subtype EP_2 _receptor expression in colon cancer tissue predicted reduced survival [2]. Seen together, it seems that induction of COX-2 is a key-factor behind progression of epithelial cell transformation to invasive cancer in colon mucosa. This logic has been practically explored evaluating the role of COX-2 inhibition on epithelial dysplasia in esophageal mucosa, although without consistent results [21]. However, understanding in part complex cell to cell interactions and signaling in composed tissues it should not be expected to be isolated single factors behind local and systemic tumor progression. Therefore, we intended to evaluate relationships between high COX-2 expression in colon cancer tissue to other factors of known importance for tumor cell division, apoptosis, and metastasis. In this perspective microarray screening should be rewarding.

With this purpose we have quantified COX-2 expression in primary tumors from 48 unselected patients with R0 resected primary tumors. Then, tumor tissue with 10 highest and lowest COX-2 expressions was chosen for subsequent hybridization in various combinations. Such cross-combinations of tissues for microarray expression analyses revealed that tumors with high intrinsic COX-2 expression displayed pronounced up-regulation of genes related to muscle fibers and cytoskeleton matrix, perhaps reflecting matrix remodeling including angiogenesis. By contrast, down-regulated genes in such tumors were more related to functional proteins and eventual cellular antigens (Table 4). Similar findings were observed in comparisons between tumor tissue and normal mucosa from the corresponding patients (Table 3). Most interesting was however, findings that normal colon tissue from patients with high intrinsic COX-2 expression in tumor tissue revealed pronounced up- and down-regulation of several gene functions. Such observations led to the conclusion that colon tissue in patients with colon cancer is significantly altered in regards to functional aspects either primarily or secondary to the appearance of manifest tumor. Analysis of significantly altered pathways at high COX-2 displayed that most alterations were related to immune reactions in the present and previous analyses [22]. This confirms our previous results based on an alternative approach where preoperative COX inhibition by indomethacin mainly affected genes and pathways that involved the immunity [22,23].

It may be rewarding to understand the control of COX-2 gene expression behind complex in vivo interactions as emphasized. According to this line we evaluated a large number of proposed genes with importance for COX-2 expression as listed in Table 6. Fourteen out of 47 genes were found to have significantly altered expression defined at least as 1.5 fold change compared to normal mucosa or by chance variation in transcript expression. Fos-B/AP1 expression displayed the most pronounced alteration of up-regulated transcripts among transcription factors. A transcription complex known to regulate COX-2 gene expression via binding to CRE-sites in the promoter region is Activator protein 1 (AP-1), which consists of homo- or heterodimers of JUN- and FOS-families with roles in several different cancers. In colorectal cancer AP-1 may be activated by either K-RAS mutation or via Wnt signals. Different genes are transcribed controlling biological activities such as proliferation, apoptosis and differentiation depending on the sub-components of AP-1 [24,25]. Six of 19 factors with emphasized importance for external cell signaling were up-regulated in tumor tissue of high intrinsic COX-2 expression (Table 7). As expected, such experiments confirmed no significant transcript with decreased expression. IL6 and protein kinas Cβ1 appeared most up-regulated in such cross hybridizations, although transcription of NF-κB was not elevated. In this context it is important to emphasize that protein and gene activation may occur despite evidence of unchanged transcription levels, usually subsequent to phosphorylation changes. Again, it was observed that 9 transcription factors and IL1β, IL6 and iNOS were significantly altered in normal colon tissue from patients with tumors of high intrinsic COX-2 expression. Individual confirmation of array results by PCR determinations displayed also a significantly higher expression of IL1β and IL6 at high COX-2. This phenomenon demonstrates that overall COX-2 expression in primary colorectal carcinoma probably includes primary and secondary alterations in tumor un-involved colon tissue.

It is well recognized that methylation of CpG island located within promoter regions of genes may silence gene expression, described as a hallmark in human cancer. This represents findings early during carcinogenesis and is maintained by DNA methyltransferase (DNMTs) [26]. Such methylation of the COX-2 promoter region may be associated with loss of gene expression as reported for gastric cancer [15,27]. Similar evidence has been reported for COX-2 gene expression in colorectal cancer [27,28], although, statistical evaluations of reported observations do not give the impression that COX-2 expression in colon cancer tissue is related to COX-2 promoter methylation. Accordingly, we did not observe significant methylation in two well-defined parts of the COX-2 promoter region in tumor and normal colon tissues. However, DNA methyltransferase was expressed at increased levels in tumor tissue compared to the corresponding normal colon tissue. Therefore, it is unlikely that promoter methylation was a significant factor behind the lack of COX-2 expression in presently investigated areas of colon cancer tissue. It remains to be determined how COX-2 is either induced in tumor tissue with intrinsic high COX-2 expression or down-regulated in tumor cells with correspondingly low overall COX-2 expression to provide the well-recognised scattered pattern of expression observed in immunohistochemical cross tissue sections. Important is our observation that COX-2 expression in normal colon tissue was also significantly increased in patients with tumors of high COX-2 expression. This points to the possibility, that the mucosa or entire colon tissue may be altered as a general phenomenon in patients with certain kind of colon tumors. Perhaps, the inflammatory activation by some kind of bacterial flora(s) could be a speculative guess.

## Conclusion

Our present study emphasized and confirmed our previous observations that increased COX-2 expression or tissue content is overall not a unanimous finding in colorectal cancer tissue. A scattered presence of COX-2 may rather represent different cell clones or different local conditions at the cellular level. Both transcription and external cell signaling factors were significantly altered as covariates to COX-2 expression in colon cancer tissue. DNA methylation of the COX-2 promoter region did not seem to be a significant factor behind COX-2 expression in either tumor tissue or normal colon tissue in the present material. Our results confirm that both local and systemic inflammation promote tumor growth and disease progression.

## Abbreviations

COX: cyclooxygenase; RIN: RNA Integrity number.

## Competing interests

The authors declare that they have no competing interests.

## Authors' contributions

AG carried out the study design, gene expression studies, DNA methylation and drafted the manuscript. HC participated in DNA methylation analysis. MA participated in extraction of RNA/DNA and carried out gene expression experiment. CL participated in study design and gene expression analysis. KKL participated in gene expression analysis. KL conceived of the study and participated in study design and helped drafted the manuscript.

All authors have read and approved the manuscript.

## Pre-publication history

The pre-publication history for this paper can be accessed here:

http://www.biomedcentral.com/1471-2407/11/238/prepub
